# Greenness, blueness, and whiteness evaluation of a quantitative nuclear magnetic resonance procedure for concurrent assay of aspirin and omeprazole in their single and fixed-dose combined tablets

**DOI:** 10.1186/s13065-025-01477-3

**Published:** 2025-05-05

**Authors:** Amal A. El-Masry, Abdallah M. Zeid, Nora A. Abdallah

**Affiliations:** 1 Department of Medicinal Chemistry, Faculty of Pharmacy, Mansoura University, Mansoura, 35516 Egypt; 2 Department of Chemistry, Michigan University, AnnArbor, MI 48103 USA; 3 Department of Pharmaceutical Analytical Chemistry, Faculty of Pharmacy, Mansoura University, Mansoura, 35516 Egypt

**Keywords:** ^1^H-qNMR, Aspirin, Omeprazole, Pharmaceutical dosage form, Greenness, Whiteness and blueness assessment

## Abstract

The Food and Drug Administration recently approved a fixed dose combination of aspirin and omeprazole that has been introduced for the treatment of gastrointestinal disorders, as it reduces the risk of upper gastrointestinal and cardiovascular adverse events in aspirin-treated patients. Therefore, an optimized eco-friendly, simple, fast, and precise quantitative nuclear magnetic resonance spectroscopy technique was presented for the concurrent estimation of that mixture in their single and combined dosage forms. The quantitative nuclear magnetic resonance spectroscopy concurrent estimation of both drugs was achieved using phloroglucinol as the internal standard and dimethyl sulfoxide as a deuterated solvent. An ideal set of acquisition parameters was determined to be 128 scans, 10 s relaxation latency, and 90° pulse angle. The selected quantitative signal of aspirin was the doublet of doublet signal appeared at 7.945 ppm, while that of omeprazole was the singlet signal at 8.18 ppm. The singlet signal at 5.69 ppm was selected for the internal standard. The spectra were subjected to integration, baseline correction, and auto phase correction. The developed quantitative proton nuclear magnetic resonance spectroscopy method was found to be rectilinear over the range of 0.05–4.0 mg mL^−1^ for both drugs. The detecting and quantifying limits for both drugs were approximately 0.01 and 0.03 mg mL^−1^, respectively. Neither labelling nor pretreatment steps were needed to assay the studied drugs using our developed quantitative nuclear magnetic resonance spectroscopy method. The proposed nuclear magnetic resonance spectroscopy approach was effectively evaluated in terms of linearity (r = 0.9999), accuracy, and precision (%RSD < 1.08). Furthermore, the suggested technique was investigated to analyze the studied drugs in their single and combined dosages. This work enables clinicians to simultaneously monitor aspirin and omeprazole levels in both single and fixed-dose combination tablets, ensuring precise dosing and effective treatment management. For patients, it supports safer therapy by reducing the risks associated with improper dosing or drug interactions in combination therapies. After evaluating the method's greenness, whiteness and blueness, it was determined that the suggested approach was environmentally friendly. The suggested approach was compared with the previously reported methods from both an analytical and eco-friendly perspective.

**Supplementary Information:**

The online version contains supplementary material available at 10.1186/s13065-025-01477-3.

## Introduction

**Fig. 1:**
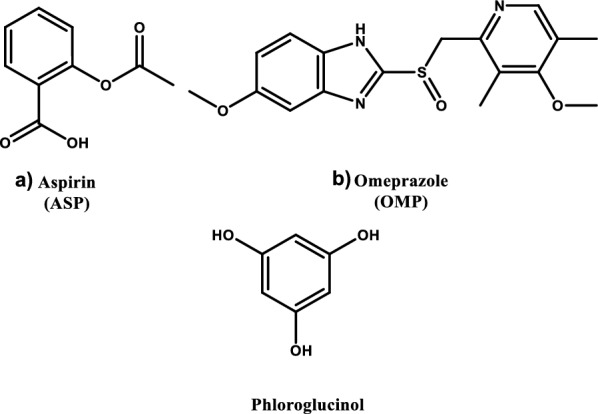
Chemical structures of the studied analytes and the internal standard

Aspirin (ASP) and omeprazole (OMP) combination has been recently approved by the Food and Drug Administration (FDA) and formulated into new pharmaceutical tablets for therapy and prophylaxis of cardiovascular disorders (CVD) in individuals suffering from stomach disorders and stomach ulcers. ASP shown in Fig. 1a, is a well-known antiplatelet medication that is thought to be essential for treating and preventing CVD [1, 2]. But, it has risky side consequences in individuals with peptic ulcers and stomach disorders [3]. Therefore, antiulcer medications must be used in conjunction with ASP to avoid that unfavorable side effect [4]. To meet such requirements, FDA approved a novel ASP and OMP combination in 2016. OMP shown in Fig. 1b, is an antiulcer that works as a proton pump inhibitor to treat the symptoms of acid reflux [5]. With this combination, patients who were at risk of developing a peptic ulcer while taking ASP should have a lower chance of stroke or heart attack [6].

Since cardiovascular disease is the leading cause of morbidity and mortality [7], various analytical methodologies were developed for concurrent estimation of ASP and OMP. A review of the literature on ASP and OMP analysis showed that various chromatographic [8–12] and spectrophotometric methods [11, 13–16] were reported for their individual or simultaneous analysis. Currently, there is no documented ^1^H-qNMR method for the concurrent analysis of ASP and OMP.

Nuclear magnetic resonance spectroscopy was initially used for structural analysis, then it has evolved to include quantification, relying on the correlation between signal strength and the number of nuclei producing resonance [17]. As a result, the ratio of the drug's signals to those of an internal standard is used to calculate the analyte's content.

NMR technique has a lot of advantages as it is characterized by the excellent precision and accuracy with the ability to efficient determination of pharmaceutical compounds purity [18]. It provides rapid, easy measurement with non-destructive analysis, and does not require pure analyte forms for calibration, as the signal strength is proportional to proton quantity [19–21]. Additionally, no prior isolation is needed for analyzing multicomponent mixtures [17, 22]. All these benefits besides the ability to recover the analytes, NMR technique is found to be a good green approach for pharmaceutical compounds analysis. Herein, we report the first green ^1^H-qNMR methodology to simultaneously evaluate ASP and OMP in their single dosage forms and fixed-dose combined tablets.

Green analytical chemistry (GAC) has appeared recently around the 2000 s [23, 24]. This field focuses on reducing hazardous reagent use and enhancing protection for both analysts and the environment [25]. Evaluating the greenness of analytical methodologies is essential for assessing their environmental impact. A comprehensive approach was used to assess sustainability, covering greenness, performance, safety, and cost-efficiency, with tools like NEMI, GAPI, eco-scale, and AGREE for greenness, RGB12 for whiteness, and BAGI for blueness. The suggested approach excels in simplicity, eco-friendliness, speed, and consistency, with no need for prior extraction.

## Experimental

### Materials and reagents

Aspirin (certified as 99.87% purity) was obtained from ADWIA Pharmaceutical Company (Qalyubia, Egypt). Omeprazole (certified as 99.80% purity) was obtained by Pharco Pharmaceuticals Company (Alexandria, Egypt).

Omez^®^ capsules, marked as containing 40.0 mg of OMP in each capsule, Jusprin^®^, marked as containing 81.0 mg of ASP per tablet and Yosprala®, labeled to contain 40.0 mg omeprazole and 81.0 mg aspirin per tablet, were bought from a local pharmacy, Mansoura, Egypt.

The deuterated solvent utilized for quantifying this mixture was DMSO-*d*_*6*_, procured from Cambridge Isotope Labs, Inc. (D, 99.96% purity). Deuterium oxide (D, 99.90%), chloroform-*d*_*1*_ (D, 99.80%), acetone-*d*_*6*_ (D, 99.90%), and formic acid were all acquired from Sigma-Aldrich. Phloroglucinol anhydrous was obtained from Chemi-pharm for Pharm. Industries in Giza, Egypt.

### Apparatus

A Bruker Avance III (AV-III-400) spectrometer was used to obtain ^1^H NMR spectra, Pharmacy Center of Scientific Excellent (PCSE), Faculty of Pharmacy, Mansoura University, Egypt. q-NMR experiments were achieved using these parameters: sample temperature (20 °C), frequency offset (6.175 ppm), spectral width (15 ppm), relaxation delay (10 s), acquisition time (4.08 s), number of scans (128), flip angle (90˚), dummy scans (2), sample spin (on), datapoints (65536), and fixed receiver gain value (32 dB). ^1^H NMR Spectra were automatically adjusted with TOPSPIN (V. 3.0, Bruker Biospin, Spring, TX, USA) for baseline and phase distortions. The energy consumption per sample was 1.0 kWh per sample.

### Preparation of standards

ASP, OMP (10.0 mg mL^−1^ each) and phloroglucinol (100.0 mg mL^−1^) were prepared individually by dissolving 0.05 g and 0.5 g of each drug raw powder, respectively, into three separate 5.0 mL volumetric flasks using DMSO-*d*_6_ as a solvent.

### Calibration curves

The prepared standards were subjected to 20 min of sonication to ensure uniformity and dissolution. Varying amounts of ASP and OMP standard solutions were accurately transferred into sealed glass vials to generate solutions spanning concentrations ranging from 0.05 to 4.0 mg mL^−1^ for both substances. A precisely measured volume of phloroglucinol (internal standard) was then introduced into each vial to produce a final concentration of 10.0 mg mL^−1^. Subsequently, the content of each vial was adjusted with DMSO-*d*_*6*_ to 2.0 mL final volume. Thereafter, q-NMR analysis was conducted by transferring 0.5 mL of each vial to a 5-mm NMR tube, followed by acquiring the proton spectra for each concentration in triplicate using optimized parameters. Calibration curves were obtained by plotting the absolute integral area ratios against the final drug concentrations of each analyte. Finally, the regression equations for the studied drugs were obtained.

### Analysis of ASP and OMP in their synthetic mixtures

ASP and OMP standards were created by combining 0.0608 g of ASP and 0.03 g of OMP in a glassy sealed vial, then topped up with DMSO-*d*_*6*_ solvent to 3.0 mL, resulting in 20.27 mg mL^−1^ and 10.0 mg mL^−1^ final concentrations for both ASP and OMP, respectively. Maintaining a ratio of 1: 2.03 between OMP and ASP replicated the medicinal ratio found in Yosprala^®^ tablets. The described procedures for generating calibration graphs were followed, and the percentage of recoveries was assessed.

### Assay of dosage forms

To assay ASP and OMP in their dosage forms, ten Jusprine^®^ tablets were taken for individual analysis of ASP, ten Omez^®^ capsules were taken for analysis of OMP, and ten Yosprala^®^ tablets (81 mg ASP and 40 mg OMP per tablet) were taken for concurrent analysis of ASP and OMP.

For the tablet dosage forms, ten tablets of each formulation (Jusprine^®^ or Yosprala^®^ formulations) were weighed, finely pulverized, and thoroughly mixed to obtain a homogenous powder. On the other hand, Ten Omez^®^ capsules were emptied and their contents were mixed well and weighed for the next steps.

Thereafter, a specific amount of each powdered drug formulation was precisely placed into an individual glass vial with stopper, and a specified volume of DMSO-*d*_*6*_ was added. To ensure complete solubility, sonication was performed for 10 min on each of the three vials. The methods described above were used to create the calibration graphs and the percentage recoveries and nominal contents of the analytes in their formulations were calculated.

## Results and discussion

**Fig. 2:**
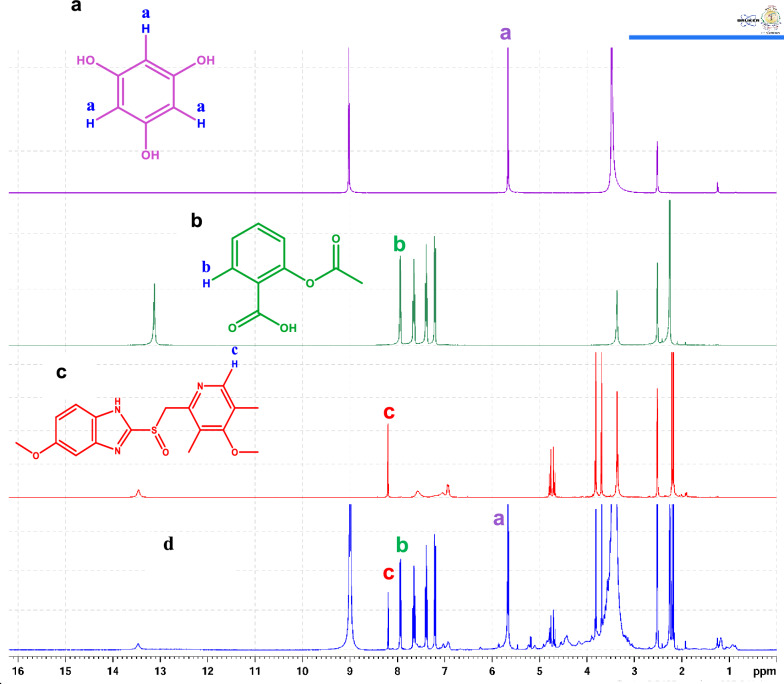
^1^H-NMR spectra of **a** phloroglucinol, **b** aspirin, **c** omeprazole, **d** mixture of aspirin, omeprazole and phloroglucinol (internal standard)

The proposed method allows for better quality control of ASP and OMP in pharmaceutical formulations, ensuring consistent drug quality for patients. Clinically, it supports improved therapeutic outcomes by enabling healthcare providers to verify the integrity of both single and combination therapies prescribed to patients. The developed ^1^H-qNMR method was thoroughly optimized to analyze the target compounds effectively in their single and combined formulations. The optimization process involved careful selection of solvent, internal standard, and exploration of various NMR's technical specifications, as pulse angle, scanning number, and relaxation delay time. DMSO-*d*_*6*_ was the optimal solvent to be used to assay the studied analytes. Phloroglucinol was identified as the ideal internal standard, producing a singlet signal at 5.69 ppm. This signal was effectively distinguished from the signals selected for quantifying ASP and OMP. To quantitatively determine the studied analytes, a doublet of doublet signal of ASP at 7.945 ppm (7.93, 7.94, 7.95, 7.96 ppm) and a singlet signal of OMP at 8.18 ppm were selected. These signals were well-resolved and conducive to qNMR application under the optimized parameters, as illustrated in Fig. 2.

### Deuterated solvents

Various deuterated solvents were assessed to identify the optimal one for achieving quantitative analysis of the two studied analytes without any interference. DMSO-*d*_*6*_, acetone-*d*_*6*_, chloroform-*d1*, and deuterium oxide were investigated in our study. DMSO-*d*_*6*_ was found as the most suitable choice due to several factors: (1) it has a signal at 2.5 ppm, which does not interfere with the signals of ASP and OMP, and (2) it did not volatilize at room temperature, rendering excellent solubility of the drugs under investigation.

### Internal standard

Several substances were investigated to choose the most suitable one to act as internal standard. Phloroglucinol was selected as the most suitable internal standard, as it showed a good solubility and stability with both ASP and OMP. Besides, its quantitative signal does not interfere with that of the studied drugs in DMSO‑*d*_*6*_. Additionally, it is available in high purity.

### Optimization of technical NMR parameters

Various parameters influencing the resolution efficiency of ^1^H-qNMR technique were investigated to determine the optimal conditions. The selected parameters were carefully chosen to guarantee the effective quantification of the studied drugs with satisfactory outcomes. These parameters encompassed the pulse angle, scan number, and relaxation delay time.

#### Scan number

**Fig. 3:**
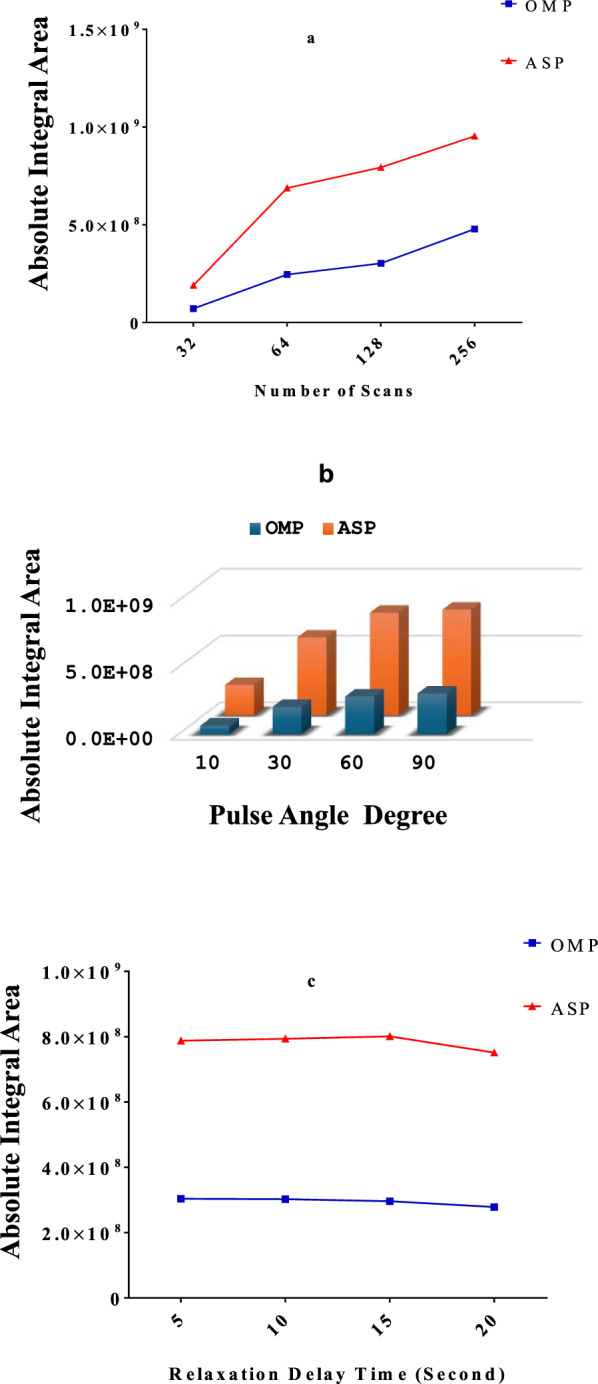
Influence of **a**: number of scans, **b**: pulse angle, and **c**: relaxation delay time, on the absolute integral area of the selected signals of omeprazole (OMP) and aspirin (ASP) in ^1^H-NMR spectra

The scanning number is a crucial parameter affecting the signal-to-noise ratio. In this study, various numbers of scans were examined including 16, 32, 64, and 128. It was observed that raising the scan number led to longer scanning times and enhanced sensitivity. Every experiment was conducted 3 times, and the averages were summarized, as illustrated in Fig. 3a. A scan number of 128 was identified as the optimal choice, offering high sensitivity and satisfactory reproducibility.

#### Pulse angle

Various pulse angles were explored while maintaining the scan number and the relaxation delay time at 128 and 10 s, respectively. The tried pulse angles were 10°, 30°, 60°, and 90°. The absolute integral areas corresponding to these angles were plotted in Fig. 3b. The findings revealed that a pulse angle of 90° is the most suitable, as it exhibited the highest values for the studied analytes.

#### Relaxation delay time

Various relaxation delay times, including 0, 5, 10, and 20 s, were examined while maintaining the scan number at 128 and the pulse angle at 90°. Figure 3c showed that the optimal relaxation delay time was 10 s. This duration was sufficient to guarantee complete relaxation (longitudinal) between two consecutive pulses, resulting in optimal signal separation and accurate quantification of the studied analytes.

## Validation

The proposed method was assessed and validated according to ICH guidelines [26]:

### Linearity, range and quantitation and detection limits

Linear relationships were obtained upon plotting the absolute integral area ratios against drugs’ concentrations over the range of 0.05–4.0 mg mL^−1^. The resulting data were statistically analyzed to study the method performance [27]. Linear regression analysis of the data and the analytical performance of the proposed approach were presented in Table 1.

**Table 1:**
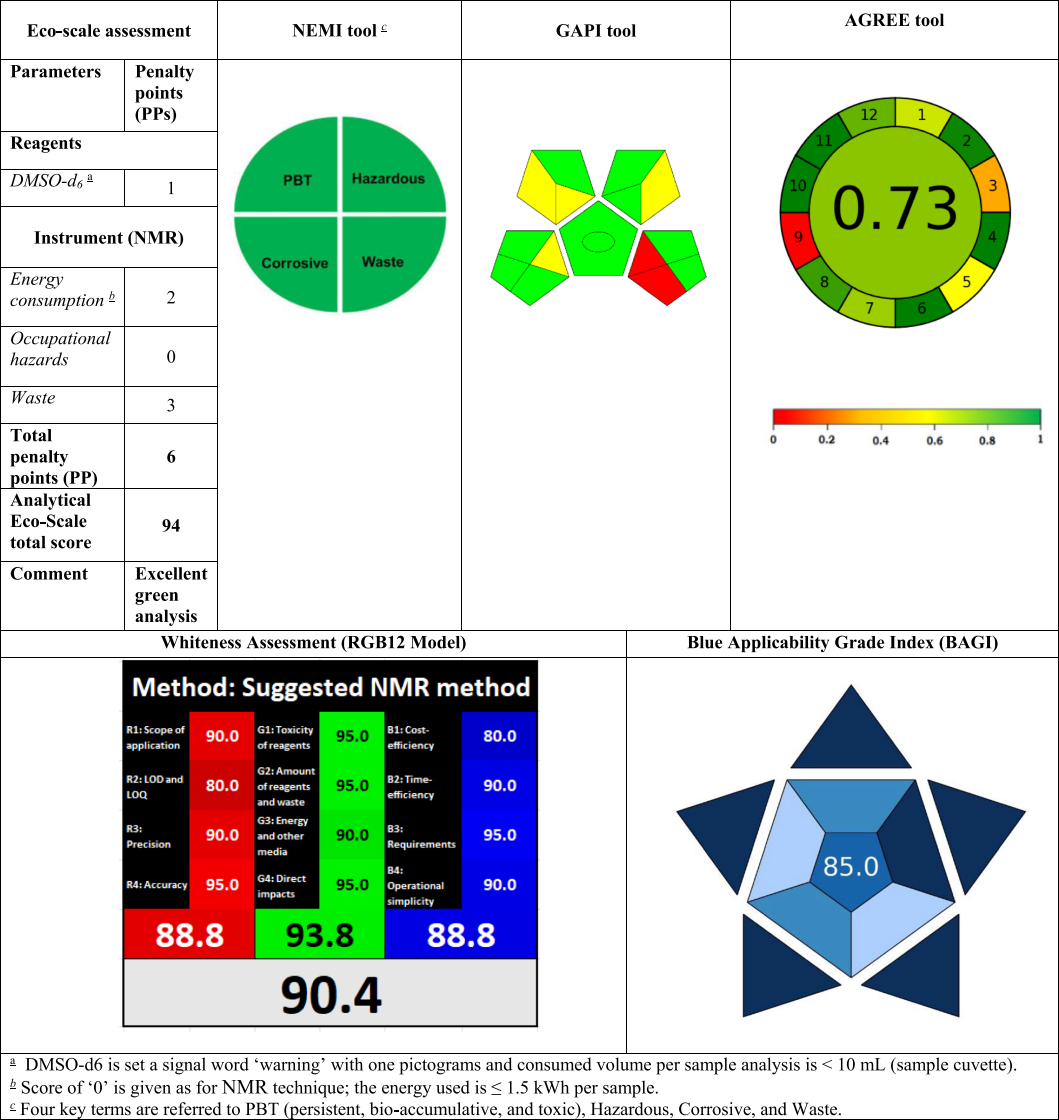
NEMI, GAPI, AGREE, BAGI, and RGB12 approaches for greenness and whiteness assessment of the proposed method

^a^DMSO‑d6 is set a signal word ‘warning’ with one pictograms and consumed volume per sample analysis is < 10 mL (sample cuvette)

^b^Score of ‘0’ is given as for NMR technique; the energy used is ≤ 1.5 kWh per sample

^c^Four key terms are referred to PBT (persistent, bio-accumulative, and toxic), Hazardous, Corrosive, and Waste

Limits of detection (LOD) and quantification (LOQ) were calculated from the regression data according to ICH [26] suggestions, using the following equations:

$${\text{LOD}}\, = \,{3}.{\text{3 S}}_{{\text{a}}} /{\text{b}}\quad \quad {\text{LOQ}}\, = \,{1}0{\text{ S}}_{{\text{a}}} /{\text{b}}$$

where,"Sa"represents the standard deviation of the intercept, and"b"denotes the slope of the calibration curve.

The results verified that the suggested method exhibited sufficient sensitivity with LOD ≤ 0.01 and LOQ ≤ 0.03 mg ml^−1^. The small values of LOD and LOQ qualifies the proposed method ideal for the concurrent assay of ASP and OMP in their co-formulated dosage form (Table S1).

### Accuracy and precision

The accuracy of the proposed NMR method was investigated. A first-order derivative spectrophotometric comparison method [14] was employed to compare the analytical results obtained by our ^1^H-qNMR method for both analytes with this reported comparison method, reporting any significant difference. In brief. three different concentrations of both drugs were measured in triplicate. The percentage of recovery was assessed for each drug concentration, and the average recovery rates (% purity) are presented in Table S2. By applying both Student *t*-test and variance ratio *F*-test, satisfactory results were obtained in terms of accuracy and precision, as there was no significant difference as shown in Table S2.

Two level precision of ^1^H-qNMR method was evaluated for both intra-day and inter-day variations by conducting triplicate assays of ASP and OMP, each in their pure form, at three concentration levels (0.5, 1.0, and 2.0 mg ml^−1^) for the studied analytes over one day and for three consecutive days, respectively. As shown in Table S3, the low relative standard deviation values attest to the method's exceptional precision and consistency employed.

### Specificity

**Fig. 4:**
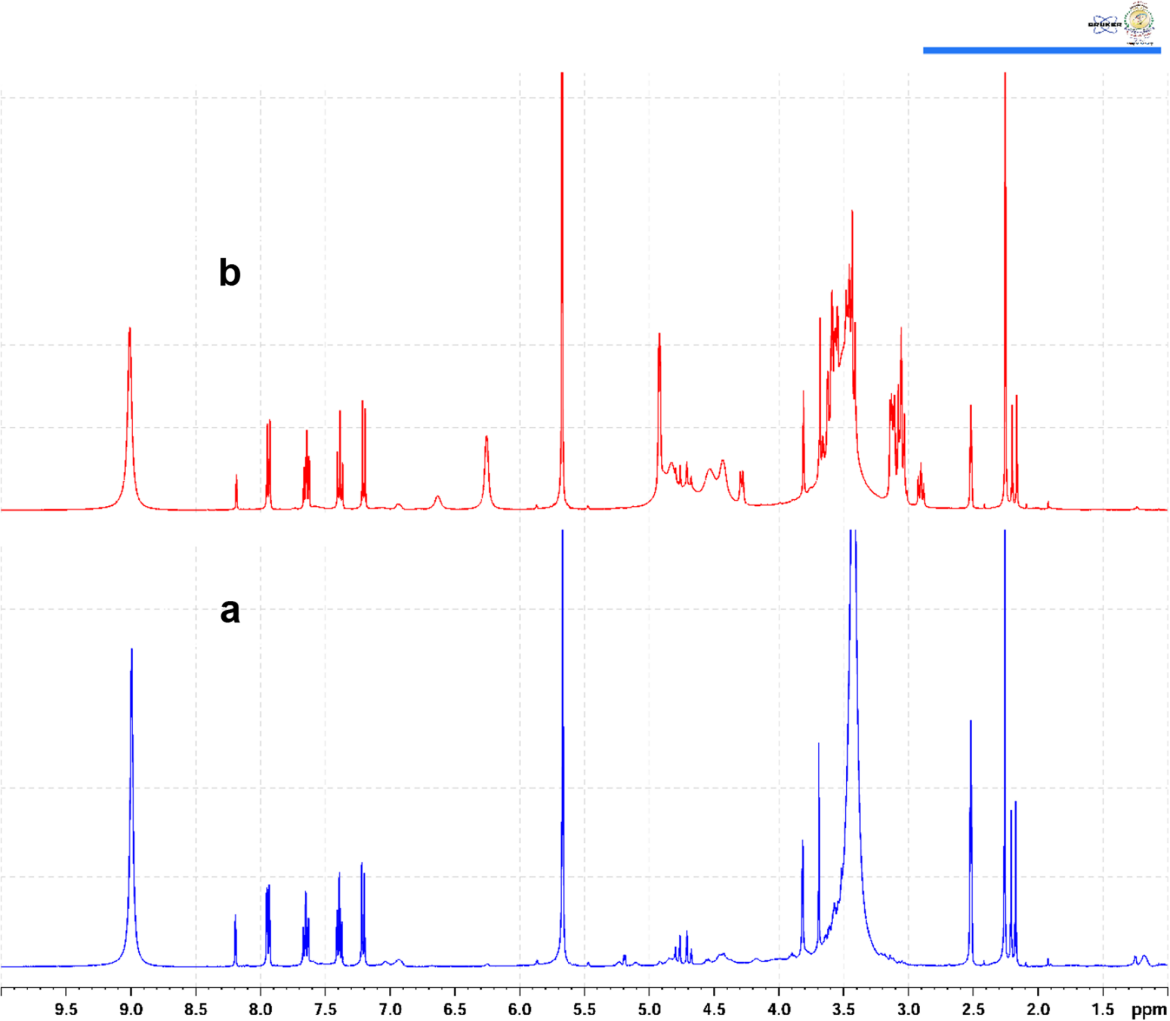
^1^H-NMR spectra of the in-lab prepared mixture of aspirin and omeprazole (**a**), and the dosage form of aspirin and omeprazole (**b**) using phloroglucinol as the internal standard in both cases

The suggested approach specificity was investigated by ensuring no interference from the excipients was detected. Proton NMR spectra of the used solvent (DMSO-*d*_*6*_), internal standard, drug samples and standards were measured separately. Figure 2 showed well-separated signals of the studied drugs and the internal standard without any overlap, demonstrating the specificity of the suggested method. Figure 4a showed ^1^H**-**NMR spectra of the synthetic mixture of ASP and OMP, while Fig. 4b showed the signals of ASP and OMP in dosage form. It was obvious that the solvent and the tablet excipients did not affect the studied drugs signals after extraction from tablet.

### Stability

The probe-head allows for the good reproducibility of repeating the experiment multiple times over a lengthy period because there is a limited contamination possibility, no dilution, or analyte-detector interaction during an NMR experiment. When utilizing NMR approach, these characteristics enable a decrease in the cost of validation measures. Drug stability was assessed by analyzing a specified sample solution at different time intervals (e.g., 0, 12, 24, and 48 h) at room temperature. The resulted relative standard deviation percentages of both drugs in Table S4 confirmed the stability of them for this time. The acquired data were consistent with the general stability rule, since the RSD was less than 3.0% and the assay difference from the original value is not greater than 1.0% [28].

## Applications

### Assay of ASP and OMP in the synthetic mixtures and in their single and combined dosage forms

The suggested ^1^H-qNMR technique was successfully applied to assay both drugs in their laboratory-prepared mixtures. Figure 4a shows the efficient resolution of the synthetic mixture of ASP and OMP with no interference or overlap between each other. Table S5 presents the comparative data obtained from the concurrent analysis of ASP and OMP in a synthetic mixture using both the recommended ^1^H-qNMR method and the comparison technique. The results showed no considerable difference in terms of accuracy and precision.

Moreover, the proposed method was efficient in assaying both drugs in single and combined dosage forms with high specificity and selectivity. Figure 4b shows the efficient separation of the ASP and OMP in their combined dosage form with no overlap from formulations'additives. The resulting data underwent statistical analysis using student's *t*-test and variance ratio *F*-test (Table S6). As detailed in Table S6, the small % RSV and high percentage of recoveries confirmed the appropriate analytical utility of the recommended technique for quality control of ASP and OMP in both single and combined pharmaceutical dosage forms.

## Evaluation of greenness, whiteness and blueness

The first investigated greenness evaluation tool for our developed method is analytical eco-scale [29], in which we subtracted 1 penalty points for DMSO‑*d*_*6*_, 2 for energy consumption, and 3 penalty points for waste generation, to render a total score of 94.0, confirnming the excellent green methodology (Table 1).

The second evaluating tool in our study is the National environmental method index (NEMI), which is an old tool [30]. The assessment result indicated the greenness of our method as it acquired green color of the four (Table 1).

Green Analytical procedures Index (GAPI) is the third greenness tool that was employed to assess the sustainability of our developed method [31, 32]. This tool is an excellent semi-quantitative tool for laboratory practice since it provides details on all 15 parameters involved in the process, from sampling to the final determination. Płotka-Wasylka and Wojnowski introduced complexed GAPI, and they also offer highly useful software to build the complexed GAPI figure automatically and with ease based on each method's inputs [33]. Complex GAPI pictogram for the proposed method that ensures its greenness is presented in Table 1.

Analytical GREEnness (AGREE) metric was also employed to assesss the greenness of our developed method [34–37]. AGREE allowed the weighing of each evaluated criterion, resulting in a total AGREE score (0–1) that represents to what extent the method is green. The proposed approaches acquire 0.72 total score (Table 1).

The Red–Green–Blue (RGB) model, which was presented lately, is thought to be a quantitative assessment metric that determines how white the analytical procedure is [38, 39]. It uses the three basic colors including red, green, and blue to represent the three essential elements of the analytical process that is being assessed. Analytical efficiency is denoted by red; adherence to green chemistry principles is denoted by green; and practical/economic efficiency is denoted by blue. The suggested method gained a total score of 97.7, indicating that they were well-fitting to the three essential principles (Table 1).

A tool called Blue Applicability Grade Index (BAGI) [40] was just released. It offers a quantitative way to evaluate the"blueness"or practicality of the analytical methodologies. BAGI score has a range of 25–100, and the most practical method is the one of the highest scores (near 100). It can be used to quickly find the strong and weak points of a method in terms of its applicability. Table 1 shows that the suggested procedure achieved a high BAGI score of 80, indicating exceptional blueness. Such a great result confirms that our method has a lot to offer in terms of general functioning, hazard mitigation, and time and money savings.

## Comparison between the proposed and the reported methods

Upon comparing the sustainability and the analytical performance of our developed method with the previously published methods for the estimation of the same binary mixture, we found that the suggested technique was simpler, greener and needed shorter time of analysis (Table 2). Regarding the simplicity, the suggested technique was the simplest, as it consumed the lowest volume of only one solvent and consequently the minimal volume of waste was generated, while most previously published methods employed large volume of two or more solvent. From the greenness point of view, our developed methodology was one of the greenest methodologies because most of the reported methods consumed large amounts of the solvents (mostly the hazardous methanol and acetonitrile). Also, some methods used hazardous solvents like toluene and chloroform which are harmful to both the analyst and the environment. While NMR spectroscopy may have lower throughput compared to high-throughput techniques such as mass spectrometry, it offers unparalleled structural and dynamic information that many other methods cannot provide. Although its operational costs can be higher, the ability of NMR to deliver comprehensive, non-destructive analysis and precise molecular characterization often justifies the investment, especially in applications where detailed molecular insights are critical.

**Table 2:**
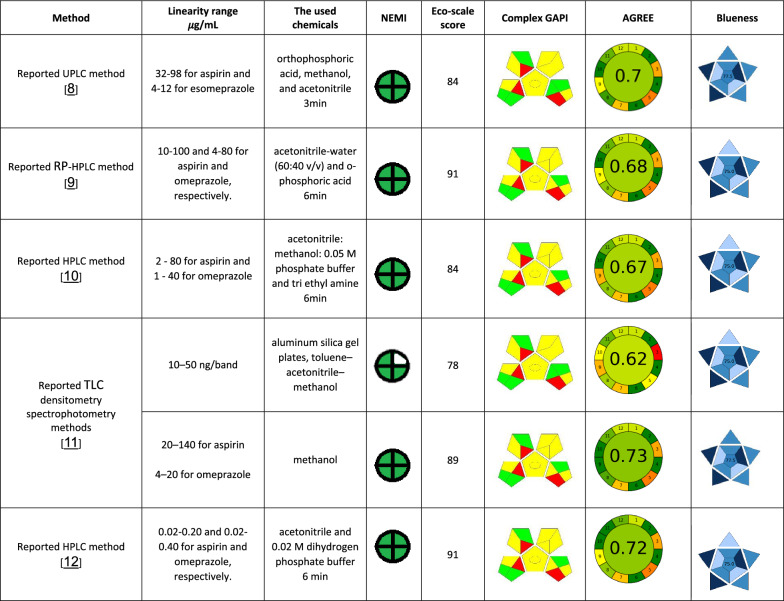

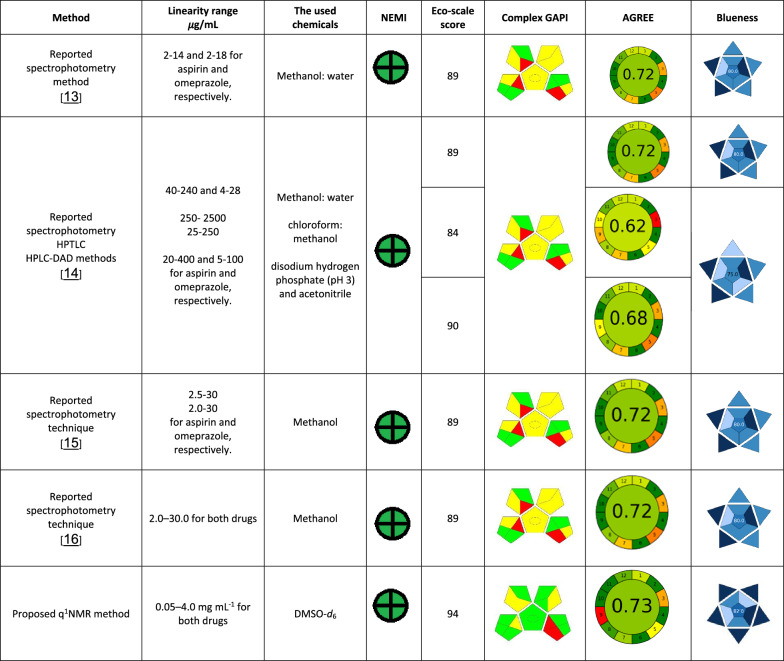
Greenness, blueness, and whiteness comparison between our proposed method and various reported methods

## Conclusion

An efficient, eco-friendly, fast, precise, and dependable ^1^H-qNMR technique was designed for the concurrent determination of a recent FDA approved binary combination of ASP and OMP. The presented method represents the first application of the non-destructive ^1^H-qNMR technique for the concurrent analysis of ASP and OMP. Validation of the suggested method was achieved in compliance with ICH recommendations. It is effectively employed to analyze ASP and OMP in their pure form, synthetic mixtures, and pharmaceutical dosage forms. The established method was applied for analyzing both drugs in single and combined dosage form with high %recovery and low RSD. The analyses were achieved using phloroglucinol (internal standard) and DMSO-*d*_*6*_ (solvent). The superiority of our developed method over the other reports lies in its quick analysis, flexibility in reference substance selection (no necessity for pure analytes), non-destructive capability to recover the analyte, and capability of analyzing multi-component mixtures without requiring any prior separation steps. The greenness, whiteness and blueness of the developed method were assessed, and the results indicated the exceptional greenness in terms of consuming a very small amount of solvent. These advantages have facilitated the application of ^1^H-qNMR in the QC analysis of the studied substances in their dosage forms. Moreover, it is expected to pave the way for numerous future applications, including pharmacokinetic studies, forensic and environmental analyses.

## Supplementary Information

Supplementary material 1

## Data Availability

No datasets were generated or analysed during the current study.
