# Supplementary material for: Greenness, blueness, and whiteness evaluation of a quantitative nuclear magnetic resonance procedure for concurrent assay of aspirin and omeprazole in their single and fixed-dose combined tablets
Source: BMC Chem. 2025 May 5;19(1):117. doi: 10.1186/s13065-025-01477-3 (PMC12053856; doi:10.1186/s13065-025-01477-3)
Supplement: Supplementary file 1 — Supplementary material 1 [file 13065_2025_1477_MOESM1_ESM.doc]

**Greenness, blueness, and whiteness evaluation of a quantitative nuclear magnetic resonance procedure for concurrent assay of aspirin and omeprazole in** **their single and fixed-dose combined tablets**

Amal A. El-Masry^1^, Abdallah M. Zeid^2,3^and Nora A. Abdallah^3*^

^1^Department of Medicinal Chemistry, Faculty of Pharmacy, Mansoura University, Mansoura 35516, Egypt

^2^Department of Chemistry, Michigan University, AnnArbor, MI 48103, USA

^3^Department of Pharmaceutical Analytical Chemistry, Faculty of Pharmacy, Mansoura University, Mansoura 35516, Egypt

^*^Correspondance : noraabdallah91@mans.edu.eg

Supplementary Material

| **Item** | **Page** |
| --- | --- |
| **Table S1:** Performance data for the determination of the aspirin and omeprazole through the laboratory performed ^1^H-qNMR method…………............................................................................................................. | S3 |
| **Table S2:** Comparative analytical data for determination of aspirin and omeprazole in pure form by the proposed ^1^H-qNMR method and comparison method ……………………………………… | S4 |
| **Table S3:** Intra-day and inter-day precision data for the assay of aspirin and omeprazole by the proposed ^1^H-qNMR method.………………………………..……………………………………...………. | S5 |
| **Table S4:** Sample stability data for the assay of omeprazole (1.0 mg/mL) and aspirin (1.0 mg/mL) by the proposed ^1^H-qNMR method.………………………………………………………..… | S6 |
| **Table S5:** Comparative resultant data from simultaneous determination of aspirin and omeprazole in their laboratory prepared mixture by the proposed ^1^H-qNMR and comparison method.……………… | S7 |
| **Table S6:** Comparative statistical analyses of the resulted data gathered from the proposed and comparison methods for determination of aspirin and omeprazole in their single dosage forms and fixed-dose combined tablets... | S8 |

**Table S1:** Performance data for the determination of the aspirin and omeprazole through the laboratory performed ^1^H-qNMR method.

| **Parameter** | **OMP** | **ASP** |
| --- | --- | --- |
| **Concentration range (mg mL^-1^)** | 0.05 – 4.0 | |
| **Correlation coefficient** | 0.9999 | |
| **Slope** | 0.14 | 0.37 |
| **Intercept** | -12 × 10^-4^ | -22 × 10^-4^ |
| **LOD (mg mL^-1^)** | 0.01 | 0.01 |
| **LOQ (mg mL^-1^)** | 0.03 | 0.03 |
| **S_y/x_** | 9× 10^-4^ | 24 × 10^-4^ |
| **S_a_** | 5× 10^-4^ | 13 × 10^-4^ |
| **S_b_** | 2× 10^-4^ | 6 × 10^-4^ |
| **% RSD** | 1.08 | 1.41 |
| **% Error = (SD / √n)** | 0.38 | 0.50 |

**Table S2:** Comparative analytical data for determination of aspirin and omeprazole in pure form by the proposed ^1^H-qNMR method and comparison method.

|  | **Proposed ^1^H-qNMR method** | | | | **Comparison method** |
| --- | --- | --- | --- | --- | --- |
| **Drug** | **Amount taken**  **(****mg mL^-1^)** | **Amount found**  **(mg mL^-1^)** | | **% Recovery^a^** | **% Recovery^a^** |
| **ASP** | 0.05 | 0.05 | | 98.00 | 101.10 |
|  | 0.10 | 0.10 | | 103.00 | 98.99 |
|  | 0.50 | 0.50 | | 100.20 | 99.31 |
|  | 0.75 | 0.75 | | 99.60 | 100.77 |
|  | 1.00 | 1.01 | | 100.60 |  |
|  | 2.00 | 1.99 | | 99.40 |  |
|  | 3.00 | 3.01 | | 100.23 |  |
|  | 4.00 | 4.00 | | 100.03 |  |
| $\bar{\text{X}}$ $\pm$ SD | 100.13 ± 1.41 | | | | 99.88 ± 1.05 |
| t-test | 0.11 (2.23)^b^ | | | | |
| *F*-value | 1.80 (8.89)^b^ | | | | |
| **O MP** | 0.05 | | 0.05 | 98.00 | 98.00 |
|  | 0.10 | | 0.10 | 98.00 | 98.40 |
|  | 0.50 | | 0.50 | 100.60 | 100.22 |
|  | 0.75 | | 0.75 | 99.87 | 99.56 |
|  | 1.00 | | 1.01 | 100.80 |  |
|  | 2.00 | | 1.99 | 99.45 |  |
|  | 3.00 | | 3.01 | 100.20 |  |
|  | 4.00 | | 4.00 | 99.98 |  |
| $\bar{\text{X}}$ $\pm$ SD | 99.61 ± 1.08 | | | | 98.78 ± 1.03 |
| *t*-test | 0.87 (2.23)^b^ | | | | |
| *F*-value | 1.11 (8.89)^b^ | | | | |

^a^ Each result is the mean recovery of three separate determinations.

^b^ Figures between brackets are the tabulated *t* and F-values at (P= 0.05).

| **Parameters** | | **OMP concentration**  **(mg mL^-1^)** | | | **ASP concentration**  **(mg mL^-1^)** | | |
| --- | --- | --- | --- | --- | --- | --- | --- |
|  |  | **0.5** | **1.0** | **2.0** | **0.5** | **1.0** | **2.0** |
| **Intra-day** | **% Found^a^** | 100.56 | 100.79 | 99.43 | 100.14 | 100.62 | 99.38 |
|  |  | 100.50 | 99.36 | 98.93 | 98.51 | 99.54 | 99.11 |
|  |  | 101.78 | 101.50 | 100.36 | 99.59 | 100.89 | 100.32 |
|  | $\bar{\text{X}}$ | 100.95 | 100.55 | 99.57 | 99.41 | 100.35 | 99.60 |
|  | **± SD** | 0.73 | 1.09 | 0.72 | 0.83 | 0.71 | 0.64 |
|  | **% RSD** | 0.72 | 1.08 | 0.73 | 0.83 | 0.71 | 0.64 |
|  | **% Error** | 0.42 | 0.63 | 0.42 | 0.48 | 0.41 | 0.37 |
| **Inter-day** | **% Found^a^** | 100.56 | 100.79 | 99.43 | 100.14 | 100.62 | 99.38 |
|  |  | 101.64 | 98.29 | 97.86 | 97.97 | 98.19 | 97.76 |
|  |  | 100.21 | 98.14 | 100.36 | 98.46 | 99.54 | 97.62 |
|  | $\bar{\text{X}}$ | 100.80 | 99.07 | 99.21 | 98.86 | 99.45 | 98.25 |
|  | **± SD** | 0.75 | 1.50 | 1.26 | 1.14 | 1.22 | 1.0 |
|  | **% RSD** | 0.74 | 1.50 | 1.27 | 1.14 | 1.22 | 1.0 |
|  | **% Error** | 0.43 | 0.87 | 0.74 | 0.66 | 0.71 | 0.57 |

**Table 3** Intra-day and inter-day precision data for the assay of aspirin and omeprazole by the proposed ^1^H-qNMR method.

^a^ Each result is the mean recovery of three individual determinations.

**Table S4:** Sample stability data for the assay of omeprazole (1.0 mg/mL) and aspirin (1.0 mg/mL) by the proposed ^1^H-qNMR method.

| **Percent assay of the sample solution** | | **Time (hour)** | |
| --- | --- | --- | --- |
| **ASP (%)** | **OMP (%)** |
| 100.60 | 100.80 | | **0** |
| 99.87 | 99.95 | | **12** |
| 100.23 | 101.11 | | **24** |
| 99.57 | 99.23 | | **48** |
| 100.07 | 100.27 | | **Mean ($\bar{\text{X}}$)** |
| 0.45 | 0.85 | | **RSD %** |

**Table S5:** Comparative resultant data from simultaneous determination of aspirin and omeprazole in their laboratory prepared mixture by the proposed ^1^H-qNMR and comparison method.

| **Drug** | **Proposed ^1^H-qNMR method** | | | | **Comparison method** |
| --- | --- | --- | --- | --- | --- |
|  | **Amount taken (mg.mL^-1^)** | **Amount found (mg.mL^-1^)** | **% Recovery^a^** | | **% Recovery^a^** |
| **OMP** | 0.5 | 0.50 | 100.21 | | 99.31 |
|  | 1.0 | 1.01 | 100.79 | | 99.87 |
|  | 1.5 | 1.49 | 99.55 | | 98.05 |
| $\bar{\text{X}}$ $\pm$ SD | 100.18 ± 0.62 | | | | 98.68 ± 0.93 |
| *t*-test | 1.71 (2.78)^b^ | | | | |
| *F*-value | 2.26 (19)^b^ | | | | |
| **ASP** | 1.02 | 1.02 | | 100.62 | 98.56 |
|  | 2.03 | 2.01 | | 99.24 | 100.98 |
|  | 3.05 | 3.05 | | 100.32 | 100.98 |
| $\bar{\text{X}}$ $\pm$ SD | 100.06 ± 0.72 | | | | 99.77 ± 1.40 |
| *t*-test | 0.13 (2.78)^b^ | | | | |
| *F*-value | 3.71 (19)^b^ | | | | |

^a^Each result is the mean recovery of three separate determinations.

^b^Figures between brackets are the tabulated *t* and *F*-values at (*P*= 0.05).

**Table S6:** Comparative statistical analyses of the resulted data gathered from the proposed and comparison methods for determination of aspirin and omeprazole in their single dosage forms and fixed-dose combined tablets.

| **Drug** | | **Proposed method** | | | | | | | | | **Comparison method** | | |  |
| --- | --- | --- | --- | --- | --- | --- | --- | --- | --- | --- | --- | --- | --- | --- |
|  |  | **Amount taken (mg ml^-1^)** | | **Amount found**  **(mg ml^-1^)** | | | | **% Recovery^d^** | | | **% Recovery^d^** | | |  |
| **Omez^a^**  **(OMP)** | | 0.5 | | 0.49 | | | | 98.07 | | | 97.89 | | |  |
|  |  | 1.0 | | 0.99 | | | | 98.64 | | | 98.48 | | |  |
|  |  | 1.5 | | 1.51 | | | | 100.50 | | | 100.12 | | |  |
| $\bar{\text{X}}$ ± SD | | 99.07 ± 1.27 | | | | | | | | | 99.01 ± 1.16 | | |  |
| *t-test* | | 0.24 (2.78)^e^ | | | | | | | | | | | |  |
| *F*-value | | 1.20 (19)^f^ | | | | | | | | | | | |  |
| **Jusprin^b^ (ASP)** | | 1.02 | | 1.00 | | | | 98.46 | | | 98.16 | | |  |
|  |  | 2.03 | | 2.01 | | | | 98.84 | | | 101.98 | | |  |
|  |  | 3.05 | | 3.05 | | | | 100.14 | | | 102.66 | | |  |
| $\bar{\text{X}}$± SD | | 99.15 ± 0.88 | | | | | | | | | 100.41 ± 2.43 | | |  |
| *t-test* | | 1.20 (2.78)^e^ | | | | | | | | | | | |  |
| *F*-value | | - 1. (19)^f^ | | | | | | | | | | | |  |
|  | **Proposed ^1^H-qNMR method** | | | | | | | | | | | **Comparison method** | | |
|  | **Amount taken (mg.mL^-1^)** | | | | **Amount found (mg.mL^-1^)** | | | | **% Recovery^d^** | | | **% Recovery^d^** | | |
| **Yosprala^®c^**  **(OMP+ASP)** | **OMP** | | **ASP** | | **OMP** | | **ASP** | | **OMP** | **ASP** | | **OMP** | **ASP** | |
|  | 0.5 | | 1.02 | | 0.49 | | 0.99 | | 97.93 | 97.92 | | 97.35 | 97.00 | |
|  | 1.0 | | 2.03 | | 0.99 | | 2.01 | | 99.36 | 98.97 | | 98.33 | 98.13 | |
|  | 1.5 | | 3.05 | | 1.47 | | 3.04 | | 98.12 | 99.77 | | 99.20 | 98.58 | |
| $\bar{\text{X}}$ $\pm$ SD | **OMP** | | | | | **ASP** | | | | | | 98.28 ± 0.93 | 97.79 0.81 | |
|  | 98.47 ± 0.78 | | | | | 98.89 ± 0.93 | | | | | |  |  |  |
| *t*-test | 0.25 (2.78)^e^ | | | | | 1.38 (2.78)^e^ | | | | | |  | | |
| *F*-value | 1.42 (19)^f^ | | | | | 1.30 (19)^f^ | | | | | |  | | |

- **^a^** Omez labeled to contain 40 mg of OMP in each capsule.
- **^b^** Jusprin labeled to contain 81 mg of ASP in each tablet.
- ^c^ **Yosprala®** labeled to contain 40 mg of omeprazole and 81 mg of aspirin in each tablet.
- ^d^ Each result is the mean recovery of three separate determinations.
- ^e^ The tabulated t values, ^f^ the tabulated F- values; both at P = 0.05.

Reference(s):

[1] A.H. Abo-Gharam, D.S. El-Kafrawy, M.M. Abdel-Khalek, M.S. Mahrous, T.S. Belal, Spectrophotometric and Chromatographic Methods for Simultaneous Determination of Aspirin and Omeprazole, Analytical Chemistry Letters, 10 (2020) 240-262.
